# Intraperitoneal Hemorrhage Caused by a Descending Colonic Arteriovenous Malformation Requiring Emergency Surgery: A Case Report and Literature Review

**DOI:** 10.70352/scrj.cr.26-0475

**Published:** 2026-07-28

**Authors:** Kenichiro Omoto, Shunsuke Takeuchi, Yuichi Nishihara

**Affiliations:** 1 Department of Surgery, Sassa General Hospital, Nishitokyo, Tokyo, Japan; 2 Department of Surgery, Keiyu Hospital, Yokohama, Kanagawa, Japan

**Keywords:** arteriovenous malformation, colon, intraperitoneal hemorrhage

## Abstract

**INTRODUCTION:**

Gastrointestinal arteriovenous malformations (AVMs) typically present with luminal gastrointestinal bleeding because they are generally located within the mucosa or submucosa. Intraperitoneal hemorrhage caused by a colonic AVM is exceedingly rare. Herein, we report a rare case of a colonic AVM that presented with intraperitoneal hemorrhage that required emergency surgical intervention.

**CASE PRESENTATION:**

A 59-year-old man presented to a local hospital with left-sided abdominal pain. CT revealed intra-abdominal bleeding, and the patient was subsequently transferred to our institution. Although he was hemodynamically stable on arrival, repeat CT demonstrated an increase in the volume of intra-abdominal hemorrhage. Given the progression of intraperitoneal hemorrhage despite stable hemodynamic status and the absence of a definitive preoperative diagnosis, emergency surgery was performed. Upon laparotomy, approximately 600 mL of hemoperitoneum was identified. A 5-cm area of serosal disruption was observed in the descending colon, and partial colectomy with stapled anastomosis was undertaken. The postoperative course was uneventful, and the patient was discharged on POD 9. Gross examination of the resected specimen revealed an intact mucosal surface, whereas hemorrhage had extended to the serosal surface. Histopathological examination demonstrated irregularly dilated arteries and veins with arteriovenous shunts extending from the deep submucosa to the muscularis propria, confirming the diagnosis of AVM.

**CONCLUSIONS:**

We report a rare case of intraperitoneal hemorrhage originating from a colonic AVM. In this case, bleeding from an AVM extending from the submucosa to the muscularis propria penetrated the serosal surface, resulting in intraperitoneal hemorrhage.

## Abbreviations

AVM
: arteriovenous malformation
DM
: diabetes mellitus
IVR
: interventional radiology
TAE
: transcatheter arterial embolization

## INTRODUCTION

AVMs are a type of vascular malformation characterized by an abnormal shunt between arteries and veins without an intervening capillary bed.^1,2)^ AVMs can occur in various parts of the body and may result in hemorrhage. Several cases of spontaneous intraperitoneal hemorrhage caused by AVMs of the stomach,^3)^ omentum,^4)^ and adnexa^5,6)^ have been reported. Colonic AVMs typically bleed into the intestinal lumen and therefore usually present with gastrointestinal bleeding rather than intraperitoneal hemorrhage. Herein, we report a rare case of colonic AVM that presented with intraperitoneal hemorrhage that required emergency surgery and provide a review of the relevant literature.

## CASE PRESENTATION

A 59-year-old Japanese man initially presented to another hospital with left-sided abdominal pain. Contrast-enhanced CT revealed intraperitoneal hemorrhage. Because bleeding from the small intestine was suspected, the patient was transferred to our hospital for further evaluation and treatment. The initial contrast-enhanced CT scan showed no definitive radiological findings suggestive of an AVM, such as abnormal vascular structures, a vascular nidus, early venous enhancement, or active contrast extravasation (**Fig. 1**). His medical history was significant for DM and dyslipidemia. He had no history of anticoagulant therapy or trauma. His father had a history of prostate cancer, and his mother had gastric cancer. On admission to our emergency department, the patient complained of abdominal pain and nausea. His blood pressure was 136/92 mmHg. Initial laboratory investigations revealed a hemoglobin level of 12.2 g/dL, hematocrit of 37.1%, platelet count of 27.2 × 10^4^/μL, white blood cell count of 10230/μL, and C-reactive protein level of 0.03 mg/dL.

**Fig. 1:**
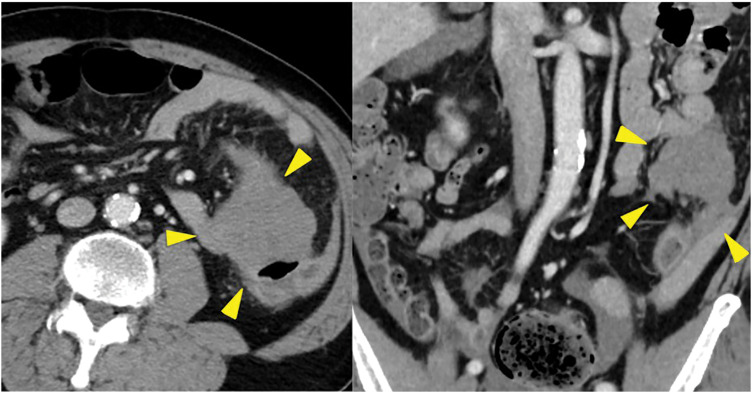
Contrast-enhanced CT demonstrating intraperitoneal hemorrhage without evidence of active contrast extravasation or free intraperitoneal air. A hematoma-dominant soft-tissue density was identified adjacent to the small intestine and descending colon (yellow arrows).

Although the patient was hemodynamically stable, repeat CT performed at our hospital demonstrated an increase in the volume of intra-abdominal bleeding, suggesting ongoing bleeding. Although hemorrhage from a tumor, such as a gastrointestinal stromal tumor, was considered in the differential diagnosis, a definitive preoperative diagnosis could not be established. Given the progressive hemorrhage despite stable hemodynamic status, emergency surgery was performed for both definitive diagnosis and hemostasis. At laparotomy, approximately 600 mL of hemoperitoneum was encountered. Serosal detachment of the descending colon, extending approximately 5 cm, was identified (yellow arrowheads) and was associated with hematoma formation and active bleeding. Partial resection of approximately 10 cm of the descending colon was performed, followed by reconstruction using a mechanical anastomosis. No abnormal findings were observed in the small intestine (**Fig. 2**). The operative time was 132 min. The postoperative course was uneventful, and the patient was discharged on POD 9. No recurrence has been observed during the follow-up period. Macroscopic examination of the resected specimen revealed no evidence of hemorrhage or neoplastic lesions on the mucosal surface (**Fig. 3**).

**Fig. 2:**
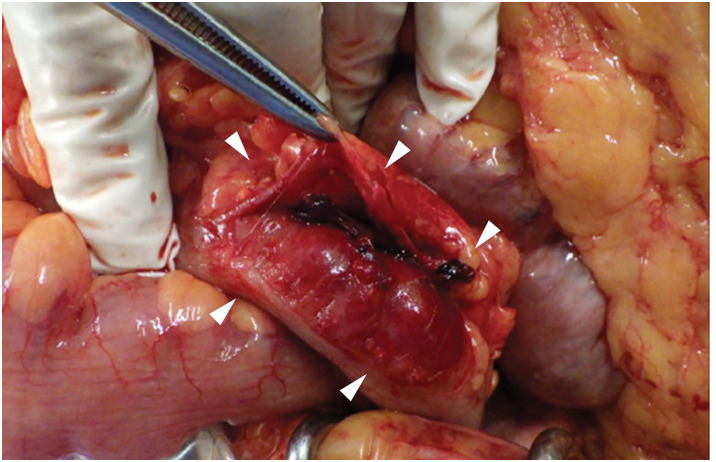
Intraoperative findings. Serosal disruption of the descending colon (white arrows), associated with hematoma formation and active bleeding.

**Fig. 3:**
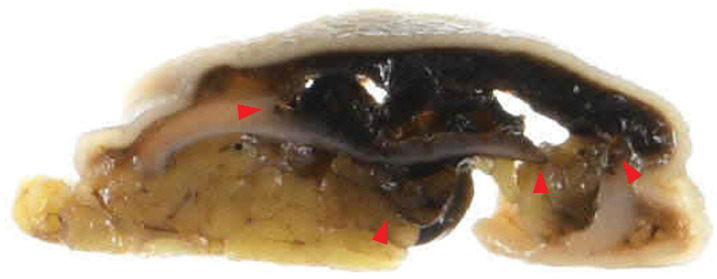
Macroscopic findings of the resected specimen. Intramural hemorrhage extending from the submucosa to the subserosa (red arrows), whereas the mucosal surface appeared grossly normal.

Histopathological examination demonstrated extensive hemorrhage from the submucosa to the subserosa, along with irregularly dilated arteries and veins extending from the deep submucosa to the muscularis propria (yellow arrowheads), confirming the diagnosis of AVM (**Fig. 4A** and **4B**). These findings suggested that hemorrhage originating from an AVM extending from the deep submucosa to the muscularis propria progressed toward the serosal surface, ultimately resulting in intraperitoneal hemorrhage.

**Fig. 4:**
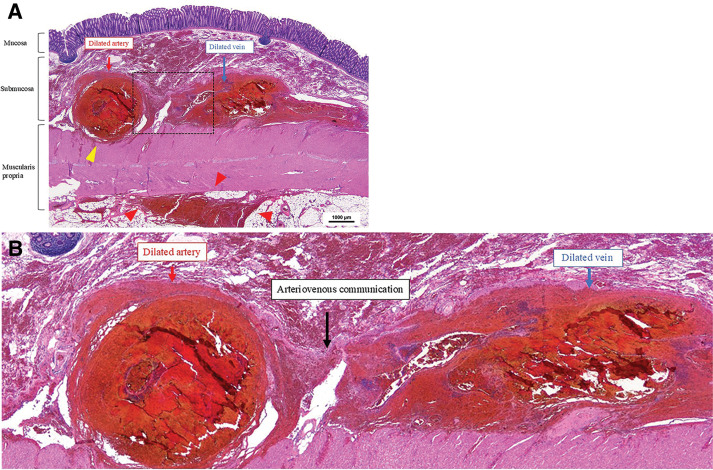
Histopathological findings of the resected specimen stained with hematoxylin and eosin. (**A**) Irregularly dilated arteries and veins extending from the deep submucosa to the muscularis propria (yellow arrowheads), consistent with an AVM. Hemorrhage extending toward the serosal surface is also evident (red arrowheads). The dashed box indicates the area shown at higher magnification in panel (**B**). (**B**) Higher-magnification view demonstrating arteriovenous communication between irregularly dilated arteries and veins (black arrow), confirming the diagnosis of an AVM. AVM, arteriovenous malformation

## DISCUSSION

According to the current International Society for the Study of Vascular Anomalies classification, AVMs are categorized as high-flow vascular malformations characterized by direct communication between arteries and veins without an intervening capillary bed.^1,7)^ AVMs can occur throughout the body, including the central nervous system, peripheral vasculature, and gastrointestinal tract.^8)^

According to the classical classification proposed by Moore et al.,^9)^ gastrointestinal AVMs are divided into 3 types. Type 1 lesions are solitary, localized vascular malformations that occur predominantly in the right colon of older patients, whereas Type 2 lesions are larger, presumably congenital lesions most commonly found in the small intestine. Type 3 lesions are associated with hereditary hemorrhagic telangiectasia. Although classical Type 1 AVMs are predominantly located in the right colon, AVMs may occur throughout the gastrointestinal tract, including the left colon.^10)^

Gastrointestinal vascular malformations are typically located within the mucosal or submucosal layers.^11)^ Consequently, they usually present with intraluminal gastrointestinal bleeding.^12)^ In contrast, intraperitoneal hemorrhage caused by a colonic AVM is extremely rare. A literature search of PubMed and Ichushi-Web identified only 3 previously reported cases of colonic AVM presenting with intraperitoneal hemorrhage.^11,13,14)^ Including the present case, only 4 such cases have been reported to date (**Table 1**).

**Table 1:** Review of published cases of intraperitoneal hemorrhage caused by colonic AVMs

Author	Year	Age/Sex	Location	Clinical presentation		Family history		Medical history		Anticoagulant therapy
Nissman	2002	74/F	Transverse colon	Right-sided abdominal pain, vomiting, diarrhea		Unknown		Mitral valve replacement		Yes
Lew	2021	24/M	Sigmoid colon	Left lower abdominal pain, fever, vomiting		None		None		No
Saigusa	2023	72/M	Descending colon	Left lower abdominal pain		None		AF, DVT		No
Present case	2026	59/M	Descending colon	Left lower abdominal pain		None		DM, DL		No
Author	Year	Hypovolemic shock	Treatment	Depth of AVM involvement			Basis of depth assessment			Outcome
Nissman	2002	Yes	Wedge resection	Muscularis propria and serosa			Histopathology			Survived
Lew	2021	No	Conservative management	Serosa			Radiological findings			Survived
Saigusa	2023	No	Partial resection with colostomy	Submucosa			Histopathology			Survived
Present case	2026	No	Partial colectomy	Submucosa and muscularis propria			Histopathology			Survived

AF, atrial fibrillation; AVM, arteriovenous malformation; DL, dyslipidemia; DM, diabetes mellitus; DVT, deep vein thrombosis

The reported patients ranged in age from 24 to 74 years, and 3 of the 4 patients were male. Among these 4 cases, 3 lesions were located in the left colon. Abdominal pain was the most common presenting symptom, and none of the patients exhibited gastrointestinal bleeding. Hypovolemic shock developed in only 1 patient. Surgical resection was performed in 3 cases, whereas conservative management was selected in 1 case. All patients experienced favorable clinical outcomes.

Among the 4 reported cases, the depth of AVM involvement was assessed using different modalities. In the present case and 2 previously reported surgically treated cases, lesion depth was determined histopathologically. In contrast, in the conservatively managed case, the depth of involvement was inferred primarily from radiological findings because no pathological specimen was available. Histopathological examination demonstrated that the lesion was confined to the submucosa in 1 surgically treated case, whereas in the remaining surgically treated cases, including the present case, the AVM extended beyond the submucosa into the muscularis propria or serosal layer. These findings suggest that deeper extension of an AVM may increase the risk of extramural rupture and subsequent intraperitoneal hemorrhage. However, this interpretation should be made with caution because the assessment of lesion depth was not based on histopathological examination in all reported cases, and the limited number of cases precludes definitive conclusions.

Treatment options for colorectal AVMs include surgical resection and IVR procedures, such as TAE following mesenteric angiography. Most available evidence regarding TAE is derived from patients with lower gastrointestinal bleeding, particularly small-intestinal vascular lesions. Superselective embolization has been reported to achieve an immediate hemostatic success rate of approximately 96%, although early rebleeding occurs in approximately 22% of patients.^15)^ Major complications, including bowel infarction, have been reported in up to 17% of cases. To the best of our knowledge, however, no previous reports have described IVR as definitive treatment for intraperitoneal hemorrhage caused by a colorectal AVM.

In the present case, although the patient remained hemodynamically stable, serial contrast-enhanced CT demonstrated progression of the intraperitoneal hemorrhage, suggesting ongoing bleeding. Furthermore, no radiological findings suggestive of an AVM, such as a vascular nidus, abnormal vascular structures, early venous enhancement, or active contrast extravasation, were identified, making selective angiography and TAE less likely to be feasible. In addition, the possibility of hemorrhage from other pathologies, including a gastrointestinal stromal tumor or other neoplastic lesions, could not be excluded preoperatively. Therefore, emergency surgical resection was considered the most appropriate approach in the present case because it allowed identification of the bleeding source, definitive hemostasis, and histopathological diagnosis. In selected patients with a clearly localized bleeding source and stable hemodynamics, IVR may represent a less invasive alternative.

## CONCLUSIONS

We report a rare case of intraperitoneal hemorrhage caused by an AVM of the descending colon. Unlike typical gastrointestinal AVMs, which usually present with luminal gastrointestinal bleeding, AVMs extending into the muscularis propria or serosal layer may result in extramural rupture and life-threatening intraperitoneal hemorrhage. Awareness of this uncommon presentation is important when evaluating patients with unexplained hemoperitoneum.

## DECLARATIONS

### Availability of data and materials

The datasets generated and/or analyzed during the current case report are available from the corresponding author upon reasonable request.
